# Development of a Fire-Retardant and Sound-Insulating Composite Functional Sealant

**DOI:** 10.3390/ma18010062

**Published:** 2024-12-27

**Authors:** Shiwen Li, Mingyu Wang, Jinchun Tu, Bingrong Wang, Xiaohong Wang, Kexi Zhang

**Affiliations:** 1 School of Materials Science and Engineering, Hainan University, Haikou 570228, China; l13631071078@163.com (S.L.); tujinchun@hainanu.edu.cn (J.T.); brwang@hainanu.edu.cn (B.W.); 2 School of Chemistry and Chemical Engineering, Hainan University, Haikou 570228, China; wangxiaohong@hainanu.edu.cn

**Keywords:** sustainable materials, building applications, composite functional filler, sealant

## Abstract

The use of traditional sealing materials in buildings poses a significant risk of fire and noise pollution. To address these issues, we propose a novel composite functional sealant designed to enhance fire safety and sound insulation. The sealant incorporates a unique four-component filler system consisting of carbon nanotubes (CNTs) decorated with layered double hydroxides (LDHs), ammonium dihydrogen phosphate (ADP), and artificial marble waste powder (AMWP), namely CLAA. The CNTs/LDHs framework provides structural support and enhances thermal stability, while the ADP layer acts as a protective barrier and releases non-combustible gases during combustion. AMWP particles contribute to sound insulation by creating impedance mismatches. The resulting composite functional sealant exhibits improved mechanical properties. In terms of flame retardancy, it boasts the lowest peak heat release rate (PHRR) of 224.83 kW/m^2^ and total smoke release (TSR) of 981.14 m^2^/m^2^, achieving the V-0 classification. Furthermore, its thermal degradation characteristics reveal a notably higher carbon residue rate. Additionally, the sound insulation capability has been significantly enhanced, with an average sound insulation level of 43.48 dB. This study provides a promising solution for enhancing the fire safety and acoustic properties of building sealing materials.

## 1. Introduction

The increasing demand for high-performance building materials has led to a growing need for sealing materials that exhibit both fire retardancy and sound insulation properties [1,2,3]. Many commercially available sealing materials fall short in possessing these crucial properties, posing substantial fire hazards and contributing to noise pollution, particularly in urban settings. Regarding these materials, research on sealants is restricted to their intrinsic performance attributes without incorporating specialized functionalities to mitigate the effects of environmental degradation. This necessitates the development of innovative materials that effectively address these challenges. When both fire and noise are present, this innovative material effectively slows the spread of fire and dramatically enhances the acoustic environment, thereby satisfying the needs of its application field. This capability is pivotal in promoting the broader adoption and expansion of its application range.

Carbon nanotubes (CNTs), known for their high thermal stability and unique acoustic wave propagation properties, significantly enhance the flame retardancy of polymer materials and exhibit the effective absorption or scattering of acoustic waves [4,5,6,7,8]. Layered double hydroxides (LDHs), as multifunctional nanomaterials with a 3D structure composed of positively charged layers and interlayer anions, offer advantages in sound insulation performance due to their abundant voids [9,10,11,12]. The addition of LDHs to polymers provides effective shielding, inhibiting heat and mass transfer under heating conditions, thereby enhancing flame retardancy [13,14,15,16]. This inherent functionality makes LDHs suitable for enhancing the sound insulation performance of resin matrices and for flame-retardant applications.

Ammonium dihydrogen phosphate (ADP) has been investigated for its flame-retardant properties [17]. Lizhuo Kong [18] demonstrated that ADP, when used as a flame retardant, undergoes thermal decomposition to produce metaphosphoric acid. This acid reacts with wood components, dehydrating them and forming thermally stable carbonized residues. Shang-Hao Liu [19] successfully incorporated ADP into an epoxy resin/bagasse composite, resulting in improved thermal stability and flame retardancy, highlighting the potential of ADP in enhancing material performance.

The waste generated from artificial marble production in industrial processes constitutes an advantageous mineral admixture, characterized by a broad particle size distribution, ease of collection, and cost-effectiveness. Artificial marble waste (AMWP), mainly composed of calcium carbonate, unsaturated polyester, and a small amount of Al (OH)_3_ and SiO_2_ [20], exhibits excellent mechanical properties, heat resistance, and stability [21,22]. Li Hai [23] addressed the issue where the incorporation of AMWP might adversely affect the fluidity and rheological characteristics of cement mortar, thereby enabling the utilization of AMWP as a partial cement replacement, yielding significant economic and environmental advantages. Tressmann D MG A [24] was utilized to augment the properties of soil pigment coatings incorporating marble waste powder, suggesting its potential application as a filler material in the realm of building sealing.

This study investigates the development of a novel composite functional sealant designed to achieve both fire retardancy and sound insulation. The approach involves growing LDH nanosheets on the CNTs skeleton to create a CNTs/LDHs frame, and the flame-retardant components ADP and organic-inorganic AMWP were incorporated to obtain CLAA composite functional fillers. The prepared composite functional fillers were then integrated into the matrix resin in a certain proportion. The thermal stability, flame retardancy, and sound insulation of the composites were studied by thermogravimetric analysis (TG), limiting oxygen index (LOI), vertical combustion (UL-94), and the B.K 4206 transfer function method. The mechanical properties and hydrophobic properties of the composite functional sealing materials were tested, and the microstructure was characterized and analyzed. This study aims to elucidate the flame-retardant mechanism and sound insulation mechanism of the composite functional sealing material.

## 2. Materials and Methods

### 2.1. Raw Materials

Carbon nanotubes (CNTs, purity > 90 wt%) were sourced from Shanghai Myriad Biochemical Science and Technology Co., Ltd. (Shanghai, China); MgCl_2_·6H_2_O (AR), Al(NO_3_)_3_·9H_2_O (AR), and ammonium dihydrogen phosphate (ADP, AR) were supplied by Shanghai McLean Biochemical Ltd. (Shanghai, China); sodium hydroxide (AR) and urea (AR) were provided by Xilong Science Co. Ltd. (Chaoshan, China); anhydrous sodium carbonate was supplied by Guangzhou Chemical Reagent Factory (Guangzhou, China). Polyacrylate emulsion (PAA) was supplied by Beijing Hongya Special Building Materials Co., Ltd. (Beijing, China); hydrophobic fumed silica nanoparticles (HFSNPs, analytically pure, specific surface area ≥ 120 m^2^/g, particle size: 30 nm) were supplied by Zhongyu Warhol New Materials Co. (Hongkong, China). The artificial marble waste (AMWP) was purchased from Hezhou, Guangxi, and the additives were purchased from Eastman Chemical Company (Kingsport, TN, USA). Deionized water was made in the laboratory.

### 2.2. Preparation of Composite Functional Sealants

**Figure 1:**
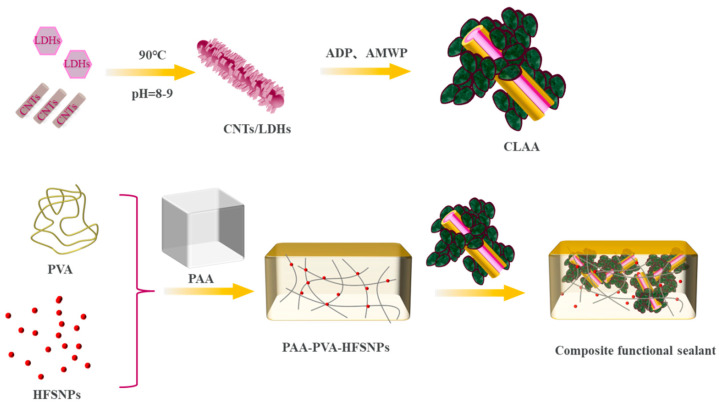
Preparation of CLAA and composite functional sealant.

As shown in Figure 1, CNTs/LDHs were synthesized via a controlled hydrothermal process. Firstly, 0.3 g of carbon nanotubes (CNTs) were introduced into a 100-milliliter solution and dispersed uniformly through mechanical stirring. Subsequently, 4.3 g of magnesium chloride hexahydrate (MgCl_2_·6H_2_O) and 2.7 g of aluminum nitrate nonahydrate (Al(NO_3_)_3_·9H_2_O) were added to the solution. After a waiting period of ten minutes, 2.6 g of urea and 1.2 g of sodium carbonate (Na_2_CO_3_) were incorporated into the mixture. The resultant solution was then subjected to a reaction at 90 degrees Celsius for 12 h, with the pH level being maintained within the range of 9 to 10 throughout the entire reaction process. Finally, the mixed solution was thoroughly washed with deionized water and subsequently vacuum-dried for 12 h to obtain the CNT/LDH composite. The obtained CNTs/LDHs with uniform composite structures were further functionalized by incorporating ADP and AMWP. The resulting composite filler, denoted as CNTs/LDHs/ADP/AMWP (CLAA), served as the key ingredient for enhancing both fire retardancy and sound insulation properties.

The preparation of the composite functional sealants involves the mixing of hydrophobic gas-phase nano-silica (HFSNPs), polyvinyl alcohol aqueous solution (PVA), and ethanol in a beaker for 20 min. Then, the polyacrylate emulsion (PAA) and additives were introduced into the mixed solution at a speed of 500 r/min, and the CLAA was added to the sealing system of 10 wt%, 20 wt%, 30 wt%, and 40 wt%, resulting in composite functional sealants recorded as A3, A4, A5, and A6, respectively. For comparison, AMWP, CNTs/AMWP(CA), and CNTs/LDHs/AMWP(CLA) were added to the sealing system to obtain composite functional sealants, labeled A1 and A2, respectively. The flame retardancy and sound insulation properties of each sample were systematically compared.

### 2.3. Characterization

Functional group properties and crystal structures were tested by Fourier transform infrared (FTIR, T27, 400–4000 cm^−1^) and X-ray diffraction (XRD, DX-2700BH, Cu Kα radiation). The micromorphologies of different samples were characterized by field emission scanning electron microscopy (SEM, Verios G4 UC microscope, Waltham, MA, USA). The chemical bonding properties of the composite functional fillers were carried out by X-ray photoelectron spectroscopy (XPS, KRATOS XSAM 800 N, Al Kα ray, HV = 1486.6 eV). Thermal stability was tested by a thermogravimetric-infrared imaging-gas chromatography in situ reaction system (TL9000) (PerkinElmer Inc., Waltham, MA, USA) in an N_2_ atmosphere, with a ramp rate of 10 °C/min, over a range of 30–800 °C. The temperature was measured by a thermogravimetric-IR-imaging-gas chromatography system (PerkinElmer Inc.), with a ramp rate of 10 °C/min. According to UL-94 test standards, vertical combustion was measured using a British FTT0082 vertical combustion tester (West Sussex, UK) according to the vertical combustion level GB/T2408, with a sample size of 130 mm × 12.8 mm × 12.8 mm. Cone calorimetry was measured using a Suzhou Yangyi Walch VOUCH 6810 cone calorimeter (Suzhou, China) with a 100 mm × 100 mm × 3 mm specimen. The combustion behavior of the composite functional sealant was investigated at a heat flow density of 35 kW, following the procedures of the ISO 5660 standard [25]. Raman testing (RAMAN) was carried out using an in-via model Raman spectrometer supplied by Renishaw, Gloucestershire, UK, with instrumental test conditions: the excitation source was a 514 nm argon ion laser line providing excitation in backscattering geometry. Sound insulation was measured by the four-sensor impedance tube method in the transfer function method of B.K 4206, and the sound insulation properties of the materials were measured according to the ASTM E2611-17 sound insulation coefficient method [26], with a frequency range of 500~6400 Hz. The mechanical properties of the materials were tested with a universal testing machine ETM105D at a speed of 50 mm/min according to GB/T5210-2006 [27]. The contact angle experiments were carried out on a Biolin Scientific contact angle meter (Gothenburg, Sweden) with a droplet volume of 5 μL. Water absorption was carried out according to ASTM D570 implementation standard [28].

## 3. Results

### 3.1. Composite Functional Filler

**Figure 2:**
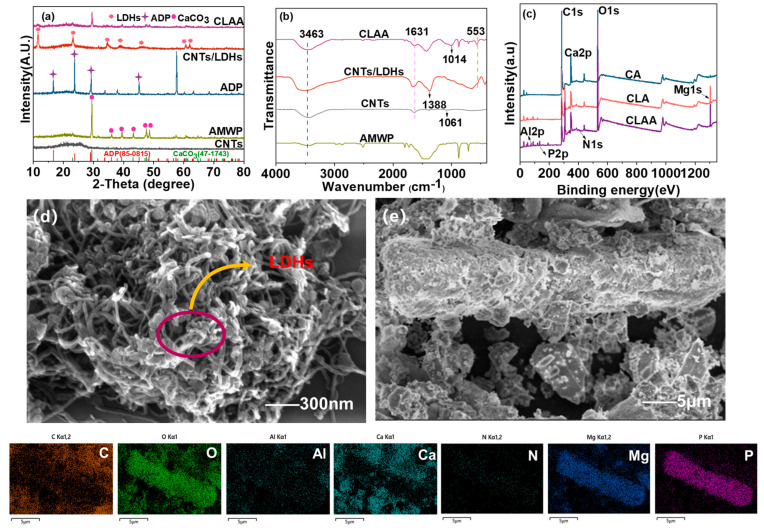
(**a**,**b**) are the XRD and FTIR spectra of CNTs, AMWP, CNTs/LDHs, and CLAA, respectively; (**c**) CA, CLA, and CLAA XPS profiles; (**d**) SEM images of CNTs/LDHs, (**e**) SEM and mapping images of CLAA.

The XRD and FTIR spectra of CNTs, AMWP, ADP, CNTs/LDHs, and CLAA are shown in Figure 2. Figure 2a,b reveals a diffraction peak of CaCO_3_ in AMWP, confirming its presence. The LDHs exhibit a series of sharp diffraction peaks at 2θ = 11.3°, 23.1°, 34.6°, 39.3°, 45.5°, 60.5°, and 62.1° [29]. This observation indicates a highly stable crystallinity in the CNT/LDH composite. Additionally, the FTIR spectrum of CLAA reveals the presence of corresponding to the asymmetric stretching vibration peak of the P-O bond (1014 cm^−1^) within the phosphate group. These findings confirm the successful loading of ADP onto the surface of CLAA. To further confirm the chemical composition of the CLAA surface, XPS analysis was performed on CA, CLA, and CLAA. As shown in Figure 2c, CLAA exhibits C1s, O1s, N1s, Mg1s, Al2p, P2p, and Ca2p levels, indicating the successful incorporation of LDHs, ADP, and CA into the composite. Notably, the Mg1s peak near 1304.3 eV, the Al2p peak near 74.1 eV [29], the N1s near 401.6 eV, and the P2p peaks near 133.1 eV, which are characteristic of LDHs and ADP, were not observed in the XPS spectra of CA or CLA, indicating that CLAA was successfully synthesized. Figure 2d reveals the unique tubular structure of CNTs and the hexagonal sheet structure of LDHs. The CNT/LDH composite, prepared by stacking LDHs on the interwoven CNTs skeleton, are tightly and uniformly wrapped by LDH nanosheets while retaining a certain degree of porosity. Upon incorporation of ADP and AMWP into CNTs/LDHs, a rod-like structure is observed in Figure 2e. This morphology is attributed to the adsorption and aggregation of phosphate ions with magnesium and aluminum ions through a combination of electrostatic attraction and surface complexation adsorption mechanisms [30,31,32,33]. The dissolution of ADP in water results in the formation of NH_4_^+^ and H_2_PO_4_^−^ ions. ADP dissolved in water may be partially adsorbed by Ca^2+^, resulting in a decrease in the amount of N detected in the solution [34].

### 3.2. Composite Functional Sealants

#### 3.2.1. SEM of Composite Functional Sealant

**Figure 3:**
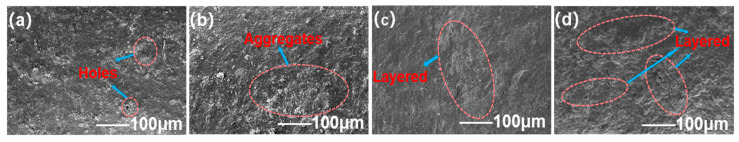
SEM images of different gelatinous layer cross sections: (**a**) A0, (**b**) A1, (**c**) A2, (**d**) A6.

The dispersion state of the composite functional filler in the matrix resin was analyzed by analyzing the microstructure of the cross-section of the composite adhesive layer using SEM. Figure 3a reveals particles in the cross-section, accompanied by noticeable voids and cracks, which may compromise the thermal barrier effect of the adhesive layer during combustion. The cross-section of Figure 3b,c shows a relatively rough structure. In addition, A1 showed obvious agglomeration, suggesting a limited dispersion of CA in the resin, which damaged the tensile properties of the composites. In contrast, the cross-section of CLA exhibits an uneven layered structure indicative of a ductile fracture structure. This improved dispersion is likely due to the synergistic effects of the CNTs/LDHs framework, ADP, and AMWP.

#### 3.2.2. Thermal Degradation Behavior

**Figure 4:**
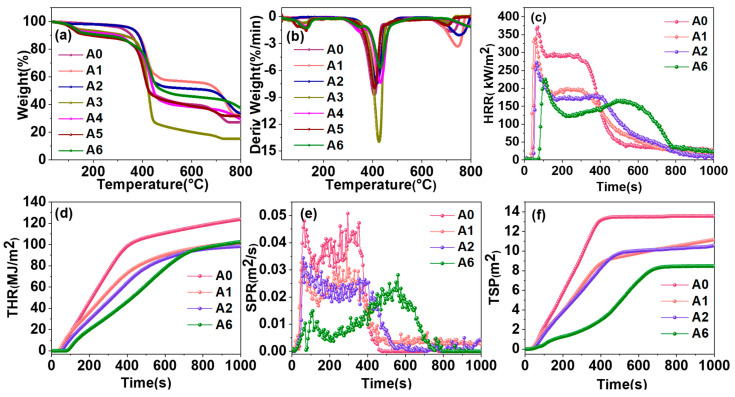
(**a**,**b**) Thermogravimetric analysis plots of samples A0, A1, A2, A3, A4, A5, A6; cone calorimetry test curves for A0, A1, A2, A6: (**c**) HRR, (**d**) THR, (**e**) SPR, (**f**) TSP.

The thermal degradation behavior of the composite functional sealant was investigated using TGA, as illustrated in Figure 4. Figure 4a shows that the thermal degradation of all composite functional sealants occurs in three distinct stages. The initial weight loss, observed between 30 °C and 240 °C, is mainly caused by the degradation of low molecular organic components within the adhesive layer and the evaporation of surface water molecules. The reduced weight loss rate of A3–A6 during this stage can be attributed to the endothermic decomposition of ADP under heating conditions. The second stage of weight loss, occurring between 240 °C and 550 °C, is mainly associated with the thermal decomposition of the matrix resin and the composite functional filler, accompanied by the release of gas. Table 1 presents the carbon residue rate after thermal decomposition, revealing an increasing trend in carbon residue content with the addition of flame-retardant treatments: A0 < A1 < A2 < A6. The DTG analysis results in Figure 4b indicate a decrease in the maximum thermal decomposition rate of A3–A6, which is attributed to the protective effects of the ADP layer on the surface of the composite functional sealant. The presence of metal oxides and phosphates improved the heat resistance of the composite functional sealant and inhibited the mass loss of the matrix resin, resulting in a lower maximum mass loss rate than the control group A0. The thermal decomposition of ADP releases phosphorus-containing free radicals and inhibits combustion generation. The metal oxides (MgO and Al_2_O_3_) formed by the thermal decomposition of LDHs act as the support and protective layer on the resin surface. The final stage of weight loss occurs in the range of 600 °C–800 °C, which is mainly caused by the decomposition of char residue [29]. Overall, A6 has the lowest mass loss rate and the best thermal stability, indicating that the introduction of CLAA effectively inhibits the rapid release of degradation products, thus reducing the maximum mass loss rate. 

#### 3.2.3. Flame Retardancy

**Figure 5:**
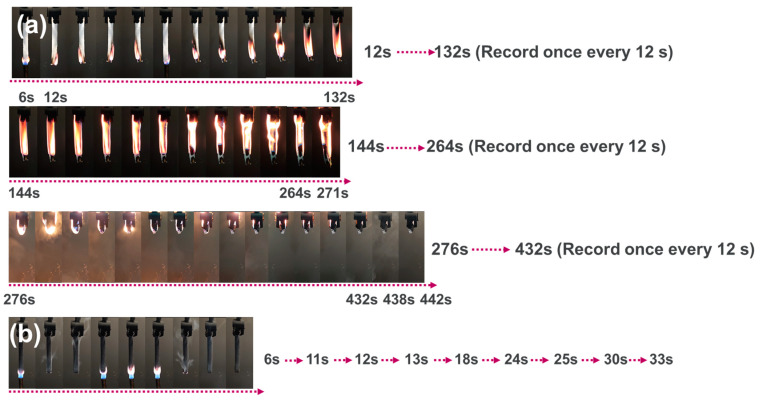
Video screenshots of UL-94 testing process: (**a**) A0, (**b**) A6.

The UL-94 test, which directly reflects the real combustion process of materials, was conducted to evaluate the flame retardancy of the composite functional sealants. As shown in Table 2, the control group A0, consisting of organic and inorganic cross-linked AMWP sealant, failed to achieve a UL-94 rating, indicating insufficient flame-retardant properties. Figure 5a further illustrates this observation, showing that A0 undergoes an intense combustion process upon ignition. The composite material begins to fall at 271 s, and the whole combustion process lasts for 442 s. After the introduction of A3–A6, the UL-94 rating gradually increased, with A6 achieving a V-0 level. The A6 sample exhibited self-extinguishing behavior, extinguishing the flame after the two ignition cycles, without any dripping during the two combustion processes. The synergistic flame-retardant effect of ADP and CNTs/LDHs contributes to the enhanced flame retardancy of the material, which is beneficial to the sealant layer. The comparison of A0 and A6 demonstrates that the introduction of CLAA can improve the flame retardancy of the composite material and give the material a certain self-extinguishing performance and anti-dripping performance.

To further study the combustion behavior of composite functional fillers, a cone calorimeter test was carried out. Analysis of Figure 4c,d reveals that A0 exhibits a significantly higher peak HRR, indicating rapid and intense combustion. With the loading of CLAA, the ignition time increases in turn. Compared with A2, A6 shows a slight decrease in PHRR by 1.20%. Table 3 also reveals that the PSPR and TSR of A0 are 0.037 m^2^/s and 1259.25 m^2^/s, respectively. This is attributed to the fact that AMWP, being an organic-inorganic composite, and the filling resin lack inherent flame-retardant characteristics. The incorporation of CLAA significantly reduced the PSPR and TSR of A6. Figure 4f illustrates that A6, which contains ADP, exhibits improved performance in inhibiting smoke release. The reduction of smoke emissions is crucial for rescue and evacuation during fire accidents.

#### 3.2.4. Analysis of Residual Char

**Figure 6:**
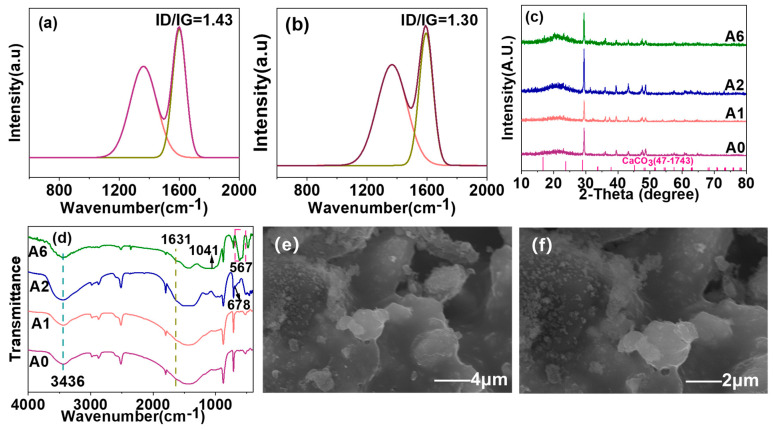
Raman spectra of residual charcoal in A0 (**a**) and A6 (**b**); (**c**,**d**) are the XRD and FT-IR spectra of the residual char. (**e**,**f**) The SEM diagram of A6 carbon residue after UL94 test.

Raman spectroscopy was used to evaluate the degree of graphitization of residual carbon, which is critical for understanding the impact of CLAA on carbon formation. It is well-established that a lower ID/IG strength ratio indicates a higher degree of graphitization and a closer stacked carbon layer. As shown in Figure 6a,b, A0 exhibits the highest ID/IG value (1.74). In contrast, the ID/IG value of A6 is 1.36, indicating an improved degree of graphitization and enhanced stability of the carbon residue layer, which is consistent with the decrease of PHRR and PSPR. In summary, the incorporation of CALL into the composite functional sealant effectively mitigates heat release and pyrolysis volatiles, thus confirming the flame-retardant ability of the sealants.

FT-IR and XRD were used to characterize the structural information of the residual char. Figure 6c shows the characteristic peak of carbon and CaCO_3_ with complete structure in AMWP, which is related to the formation of the carbon layer and the excellent stability of AMWP, which plays an indispensable role in flame retardancy. The 3436 cm^−1^ in Figure 6d is the water molecule produced by combustion, and the 1631 cm^−1^ is mainly the C=O absorption peak, which corresponds to the oxidation degree of the carbon layer of the functional sealant. The P-O stretching vibration and O-P-O bending vibration of ADP decomposition correspond to the peaks at 1041 cm^−1^ and 567 cm^−1^, respectively. Similarly, the absorption peak at 678 cm^−1^ is indicative of metal–oxygen bonds decomposed from LDHs. These observations suggest a tight integration between ADP and LDHs, which effectively collaborates to prevent the occurrence of combustion.

The char layer of A6 was characterized by SEM, as shown in Figure 6e,f. It was found that the surface of the residual char layer of CLAA has a well-defined lamellar structure that is intact. This structured layer serves as an effective barrier, preventing the escape of volatile gases from pyrolysis during combustion, and shielding the underlying material from external oxygen and heat transfer. Consequently, this impedes the heat and mass transfer during combustion, contributing to the flame-retardant effect.

#### 3.2.5. Sound Insulation Performance

**Figure 7:**
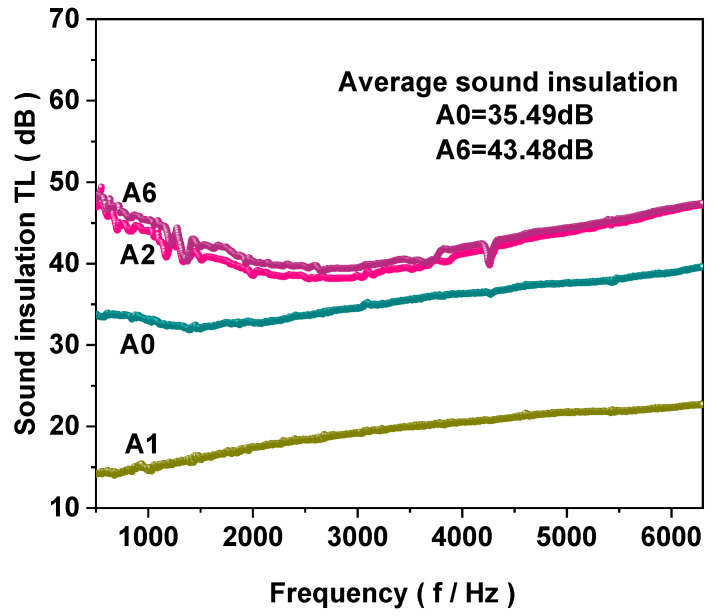
Sound insulation characteristic curve of composite functional sealant.

Based on the ASTM E2611-17 test results, the sound insulation characteristic curve of the composite functional sealant reveals specific trends, which can be explained by the acoustic properties of the materials involved and their interaction with sound waves. Figure 7 illustrates these trends, showing that at high frequencies, the sound insulation performance gradually increases with frequency. The sound insulation performance of A1 falls below that of A0, potentially because the integration of AMWP with resin is more refined. The CaCO_3_ present in AMWP efficiently modifies the surface roughness, leading to multiple reflections of the incoming sound wave. These reflections create a friction effect that transforms the sound wave energy into heat, facilitating its dissipation [35,36]. Furthermore, the rich void structure of CNTs/LDHs, in conjunction with ADP and AMWP, contributes to achieving an exceptional sound insulation effect, simultaneously enhancing the high performance of the composite functional fillers to their utmost capacity. Specifically, the average sound isolation of A6, which contains these modifications, is 43.48 dB, representing a 7.99 dB increase compared to A0, the baseline without these additions.

#### 3.2.6. Flame-Retardant Mechanism and Sound Insulation Mechanism

**Figure 8:**
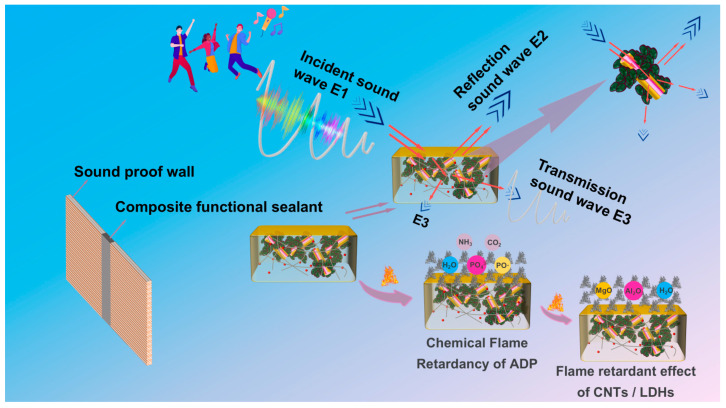
Flame-retardant mechanism diagram of composite functional sealant and propagation path diagram of acoustic wave in composite functional sealant.

The potential synergistic flame-retardant mechanism and sound insulation mechanism are detailed in Figure 8 and summarized as follows:

Within this composite, ADP plays a crucial role by emitting non-combustible gases like ammonia, thereby elevating the concentration of such gases. Additionally, the phosphate component present acts as an inhibitor, disrupting the combustion chain reaction. The incorporation of CNTs/LDHs, which boast exceptional thermal conductivity, facilitates the formation of MgO and Al_2_O_3_ while also aiding in the distribution of heat. In turn, this minimizes cracks and holes during combustion, ensuring that the sealant is preserved to the fullest extent possible.

The improvement of the sound insulation effect in composite functional sealants is primarily attributed to the incorporation of CLAA, which alters the pathway of sound wave propagation. Upon introduction of CLAA, the acoustic energy is primarily dissipated through vibrations and heat transfer associated with these particles [37]. When sound waves encounter the composite functional fillers embedded within the resin, they induce vibrations, converting a portion of the acoustic energy into mechanical energy. Simultaneously, the vibrations cause friction among CNTs/LDHs, ADP, and AMWP, further converting additional acoustic energy into heat energy and facilitating heat transfer. Consequently, embedding this composite functional filler within the resin emerges as an effective approach to augmenting sound insulation capabilities.

#### 3.2.7. Mechanical Property

**Figure 9:**
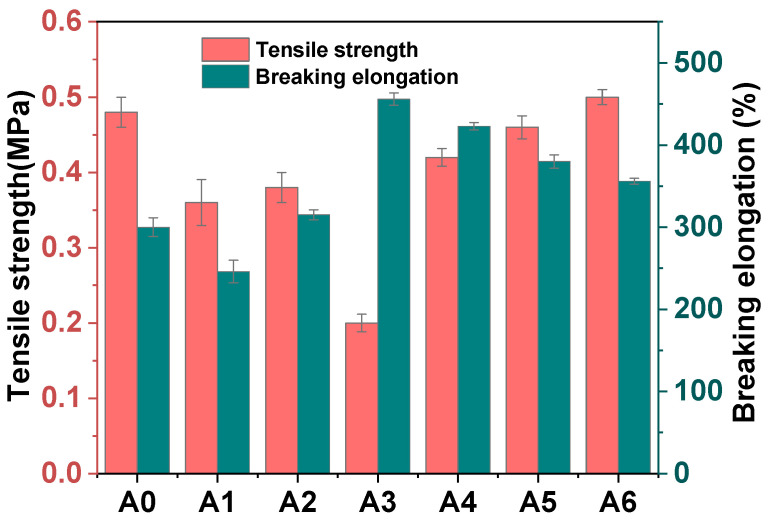
Mechanical properties of A0 to A6.

The incorporation of composite functional fillers into the resin was scrutinized to evaluate their influence on mechanical properties. Tensile tests were conducted on samples A0, A1, A2, and A6 to assess the changes in tensile strength and elongation at break. From Figure 9, it is evident that A0, A1, and A2 serve as control samples. The introduction of CLAA results in a noticeable trend of increased tensile strength and reduced elongation at the break from A3 to A6. Notably, A6 demonstrates a tensile strength of 0.5 MPa and an elongation at a break of 356%, marking a 1.04% increment in elongation compared to A0. The composite functional sealant meets the standard of high modulus sealant and can be suitable for certain deformation. From a fracture mechanics standpoint, the interplay between the intrinsic damage process and the extrinsic crack tip shielding mechanism is evident. The observed toughness is attributed to the compatibility between the composite functional filler and the resin [38,39,40]. Under stress concentration, the composite functional sealant may fail to withstand external tensile forces, resulting in the disruption of the connections between CNTs/LDHs, ADP, and AMWP. This leads to the formation of cracks in the structurally weaker areas of the sealant. As deformation intensifies, the resin and composite functional filler may undergo tearing or even fracture.

#### 3.2.8. Hydrophobic Property

The hydrophobicity of the composite functional sealant was evaluated through measurement of the water contact angle and water absorption. Table 4 presents the results, indicating that the presence of hydrophobic fumed silica nanoparticles (HFSNPs) enhances the dispersibility of the particles in the aqueous phase. Therefore, the water contact angle of A0 to A6 is greater than 90°, and the water absorption rate is maintained between 0% and 0.4%. With the increment of CLAA addition, the water contact angle increased from 93° to 110°. This increase can be attributed to two factors, on the one hand, HFSNPs reduce the surface energy of the adhesive layer and increase the surface roughness, thereby increasing the static contact angle [41]. On the other hand, due to the principle of similar miscibility, PVA and AMWP interact with HFSNPs [42]. However, the lower contact angle of A3 is due to the insufficient amount of CLAA, which results in weak rigid particle support, thereby improving the water absorption performance of the sealant.

## 4. Conclusions

By incorporating functional fillers into the matrix resin, we developed composite functional sealants with enhanced flame-retardant and acoustic insulation properties. The findings from flame retardancy and acoustic performance tests demonstrate that the composite structure of CLAA imparts superior characteristics to composite functional sealant compared to CA, CLA, and CLAA. In terms of flame retardancy, it stands out with a peak heat release rate (PHRR) as low as 224.83 kW/m^2^ and a total smoke release (TSR) of 981.14 m^2^/m^2^, qualifying it for the V-0 classification. Moreover, its thermal degradation properties show a remarkably increased carbon residue rate. Subsequently, its sound insulation capability has undergone significant improvement, boasting an average sound insulation level of 43.48 dB. The inclusion of a self-supporting and protective layer of CNTs/LDHs and ADP significantly improved the flame-retardant efficacy. Furthermore, the CNTs/LDHs and ADP, along with their decomposition-derived metal oxides (MgO, Al_2_O_3_) and phosphates, facilitate the carbonization process, forming a protective carbon layer on the resin surface. The composite functional fillers within the resin effectively slow down combustion kinetics and inhibit the combustion chain reaction through the formation of a char layer, the release of water vapor, and the impeded volatilization of phosphorus-containing radicals into the combustion zone. Additionally, the composite functional sealant contains rigid particles of organic-inorganic AMWP, which reflect acoustic waves. These reflections create a friction effect that transforms the sound wave energy into heat, facilitating its dissipation. The acoustic insulation of composite functional sealant was further enhanced due to the acoustic attenuation properties of CNTs, which interact with acoustic waves, along with the structure of LDHs that feature abundant voids.

To fulfill the sealing needs of various applications, composite functional sealants are engineered to withstand the impacts of fire and noise throughout their operational lifespan, thereby ensuring a quieter and more tranquil living space for residents. Furthermore, during industrial production, it is crucial to take into account both the cost-efficiency and environmental sustainability of these multifunctional sealants, with the aim of maximizing their practical utility in the construction sector. In summary, the versatility of sealing materials extends beyond flame retardancy and sound insulation; there is potential for further exploration and development in other functional aspects.

## Figures and Tables

**Table 1:** Thermogravimetric analysis parameters of composite functional sealants under N_2_ atmosphere.

Samples	T−_1wt_%/°C	T_max1_/°C	Residual Amount at 800 °C/wt%
A0	/	406	27
A1	/	419	33
A2	/	417	34
A3	124	425	15
A4	126	430	29
A5	128	407	31
A6	133	431	37

**Table 2:** Flame-retardant properties of A0, A1, A2, A3, A4, A5, and A6.

Samples	UL-94		
	$t_{f}$/s	Dripping	Rating
A0	>30	Yes	/
A1	0	No	V-0
A2	0	No	V-0
A3	>30	Yes	/
A4	>30	Yes	/
A5	15	No	V-1
A6	0	No	V-0

Annotation: The total residual flame time t_f_ of a group of samples adjusted in any state.

**Table 3:** Cone calorimetry test data for A0, A1, A2, A6.

Samples	A0	A1	A2	A6
TTI (s)	33	39	47	75
PHRR (kW/m^2^)	339.23	370.52	271.76	224.83
PSPR (m^2^/s)	0.037	0.050	0.034	0.024
TSR (m^2^/m^2^)	1259.25	1568.73	1192.96	981.14

**Table 4:** Contact angle and water absorption of composite functional sealants.

Samples	A0	A1	A2	A3	A4	A5	A6
Contact angle/°	103 ± 2	97 ± 3	104 ± 1	92 ± 2	95 ± 1	101 ± 2	111 ± 2
Water absorption/%	0.232 ± 0.012	0.093 ± 0.022	0.080 ± 0.054	2.360 ± 0.052	0.105 ± 0.077	0.084 ± 0.052	0.046 ± 0.02

## Data Availability

The original contributions presented in this study are included in the article. Further inquiries can be directed to the corresponding author.
